# Air Pollution and Subtypes, Severity and Vulnerability to Ischemic Stroke—A Population Based Case-Crossover Study

**DOI:** 10.1371/journal.pone.0158556

**Published:** 2016-06-30

**Authors:** Ravi Maheswaran, Tim Pearson, Sean D. Beevers, Michael J. Campbell, Charles D. Wolfe

**Affiliations:** 1 Public Health GIS Unit, School of Health and Related Research, University of Sheffield, Sheffield, United Kingdom; 2 Environmental Research Group, King’s College London, London, United Kingdom; 3 Design, Trials and Statistics Section, School of Health and Related Research, University of Sheffield, Sheffield, United Kingdom; 4 Division of Health and Social Care Research, King’s College London and National Institute for Health Research (NIHR) Biomedical Research Centre, Guy’s and St Thomas’ NHS Foundation Trust and King’s College London, London, United Kingdom; University of Missouri, UNITED STATES

## Abstract

**Background and Purpose:**

Few studies have examined the association between air pollutants and ischemic stroke subtypes. We examined acute effects of outdoor air pollutants (PM_10_, NO_2_, O_3_, CO, SO_2_) on subtypes and severity of incident ischemic stroke and investigated if pre-existing risk factors increased susceptibility.

**Methods:**

We used a time stratified case-crossover study and stroke cases from the South London Stroke Register set up to capture all incident cases of first ever stroke occurring amongst residents in a geographically defined area. The Oxford clinical and TOAST etiological classifications were used to classify subtypes. A pragmatic clinical classification system was used to assess severity. Air pollution concentrations from the nearest background air pollution monitoring stations to patients’ residential postcode centroids were used. Lags from 0 to 6 days were investigated.

**Results:**

There were 2590 incident cases of ischemic stroke (1995–2006). While there were associations at various lag times with several pollutants, overall, there was no consistent pattern between exposure and risk of ischemic stroke subtypes or severity. The possible exception was the association between NO_2_ exposure and small vessel disease stroke—adjusted odds ratio of 1.51 (1.12–2.02) associated with an inter-quartile range increase in the lag 0–6 day average for NO_2_. There were no clear associations in relation to pre-existing risk factors.

**Conclusions:**

Overall, we found little consistent evidence of association between air pollutants and ischemic stroke subtypes and severity. There was however a suggestion that increasing NO_2_ exposure might be associated with higher risk of stroke caused by cerebrovascular small vessel disease.

## Introduction

Stroke is a major cause of mortality and morbidity worldwide [1]. There is an expanding evidence base regarding the acute effects of short-term exposure to outdoor particulate and gaseous air pollutants on stroke risk [2–4]. Reported lag periods between exposure and occurrence of stroke vary, ranging from a few hours to six days [5,6].

Studies which set out to compare ischemic and hemorrhagic stroke suggest that the association between air pollutants and stroke is mainly confined to ischemic stroke [7–12]. A number of potential mechanisms have been proposed to explain the links between air pollutants and cardiovascular disease, including transient increases in blood coagulability and plaque rupture [13]. These mechanisms primarily relate to ischemic stroke.

Ischemic stroke may be subclassified by clinical and etiological subtypes [14,15]. Few studies, however, have examined the association between air pollutants and ischemic stroke subtypes and the results are mixed, with some evidence of association between some of the air pollutants investigated and ischemic stroke caused by large and small vessel disease [5,9,16,17].

A further study examined acute effects of air pollutants on mild and severe ischemic stroke and found that air pollution was only associated with mild ischemic stroke [10]. In addition, we recently observed that there is a suggestion that living in areas with elevated outdoor PM_10_ and NO_2_ concentrations might be associated with increased incidence of mild, but not severe, ischemic stroke [18].

There is also increasing interest in susceptibility to acute ischemic stroke in response to exposure to outdoor air pollution. A small number of studies have investigated a range of factors including age, gender, smoking, diabetes and preexisting cardiovascular disease, with mixed results [5,9,11,16,17].

The aim of our study was to examine the acute effects of outdoor particulate and gaseous air pollutants on subtypes and severity of incident ischemic stroke and investigate if sociodemographic factors, preexisting medical conditions and pre-stroke lifestyle factors increased susceptibility to ischemic stroke [19].

## Methods

### Study design

We used a time stratified case-crossover study design to examine the effects of outdoor air pollutants on ischemic stroke risk [20–22]. The design allows for control of individual characteristics that do not vary over short time scales as each patient acts as his or her own control. Control days were the same day of the week as the lagged exposure day (with lags of 0–6 days examined) and were selected from the same season in which the lagged exposure day occurred. Years were divided into four three-month seasons (winter, spring, summer, autumn) with winter starting in December.

### Stroke incidence data

Stroke cases were obtained from the South London Stroke Register [23]. The Register was set up in 1995 to capture all incident cases of first ever stroke occurring amongst residents in a geographically defined area of south London. The population in the Register area was 272,000 in the 2001 national population census. The geographical area was expanded in November 2004. The Register used multiple active surveillance methods for case capture. Hospital and community notification sources included accident and emergency records, hospital staff, brain imaging requests, death certificates, coroners’ records, general practitioners, community nurses and therapists, bereavement officers, social services, hospital based stroke registries, general practice computer records and notification by patients or relatives. Estimates of completeness of case capture ranged from 80 to 88% [24,25]. We used cases from 1995–2006 for this analysis including cases from the expanded Register area.

Register staff examined patients within 48 hours of notification and organized investigations using a standardized protocol which included neuroimaging, with additional investigation for ischemic stroke using an investigation algorithm incorporating carotid duplex and transcranial Doppler scanning, trans-thoracic echocardiography, trans-esophageal echocardiography and hematological investigation as appropriate [26]. The Register implemented the Oxford clinical classification system in 1995, with cerebral infarction being categorized as total anterior circulation infarct (TACI), partial anterior circulation infarct (PACI), posterior circulation infarct (POCI) and lacunar infarct (LACI) [14]. The Trial of Org 10172 in Acute Stroke Treatment (TOAST) classification of ischemic stroke subtypes based on etiology was fully implemented from 2000 [15]. Three TOAST categories were used in this analysis–large artery atherosclerosis, cardioembolism and small vessel occlusion. Previous studies examining the acute effects of air pollutants on ischemic stroke subtypes have used both these classification systems [5,9,16,17].

With regard to stroke severity, the National Institutes of Health Stroke Scale was only fully implemented in 2001 and in addition had missing information on patients who died before being admitted or assessed. We therefore used a pragmatic clinical classification system for assessing severity, which we have reported on previously [18]. In summary, a patient who had any of the following at initial assessment was classified as having sustained a severe stroke: urinary incontinence following stroke; unable to swallow following stroke; Glasgow coma score <9; or died <2 days of stroke onset. Patients who had none of the above were classified as having sustained a mild stroke. This pragmatic classification allowed all patients to be classified as mild or severe as relevant information was available from the start of the Register.

The Register also collected information on risk factors for stroke. We used data on the following risk factors to assess if patients with these risk factors were more susceptible to the adverse effects air pollutants in terms of stroke risk: sociodemographic characteristics (age, gender, social class, ethnicity); pre-existing cardiovascular conditions (hypertension, coronary heart disease, atrial fibrillation, diabetes, transient ischemic attack); and pre-stroke lifestyle-related factors (current smoker; high alcohol consumption classified as >21 units per week for men, and >14 units per week for women).

The study had approval from the ethics committee of Guy’s and St Thomas’ Hospital Trust, King’s College Hospital, London. Written informed consent was obtained from patients or next of kin for participation in the South London Stroke Register. The data extract for this study was pseudoanonymised prior to analysis.

### Exposure data

Air pollution data were obtained for particulate matter less than 10 microns in diameter (PM_10_), nitrogen dioxide (NO_2_), ozone (O_3_), carbon monoxide (CO) and sulfur dioxide (SO_2_) from outdoor background air pollution monitoring stations in London. Hourly measurement data were obtained from all monitoring stations and daily averages were calculated where there were hourly values for 18 or more hours per day. All stations were managed by the Environmental Research Group at King’s College, London with calibration controls and validity checks carried out before release of data for analysis. Distances from monitoring stations were calculated using the grid references of monitoring stations and patients’ residential postcode centroids. An increasing number of monitoring stations came into operation over the 12-year study period. Pollution concentrations used were those from the nearest monitoring station with a valid reading for the pollutant examined on the relevant day.

Hourly temperature measurements were obtained from London Heathrow meteorological station and a daily average computed when there were 18 or more hourly values per day. National weekly influenza rates, based on general practice consultations for influenza-like illness, were obtained from the Research and Surveillance Centre of the Royal College of General Practitioners.

### Statistical analysis

Statistical analysis was carried out using conditional logistic regression in Stata, with analyses stratified by patient. Air pollutant effects were investigated using lags from 0 to 6 days and an average for the 0–6 day period. The average was calculated when there were values for five or more days. Models were run for all ischemic strokes combined and for each of the subtypes and severity categories. Temperature was modeled using natural cubic splines. Influenza rate was entered as a continuous variable. Effect measure modification of the exposure-outcome relationship by sociodemographic factors, lifestyle-related risk factors and pre-existing medical conditions was examined using all ischemic stroke and the 0–6 day average pollutant values. Results are presented as odds ratios with 95% confidence intervals (CI) for an inter-quartile range increase in pollutants. We used the inter-quartile range of the dependent variable as the unit for estimation of an effect since it facilitates comparison of exposure-outcome associations across a range of pollutants measured using different units. This approach has also been used by others [5,10,11]. Given the inter-quartile range, it is straightforward to change the estimate to standard units.

Sensitivity analyses involved using control days sampled from the same calendar month instead of the same season, and using pollution values obtained from the same monitoring station for an individual patient in case there were was variation introduced by using values from different monitoring stations.

## Results

There were 2590 incident cases of ischemic stroke recorded on the Stroke Register from 1995–2006. The characteristics of these patients are shown in Table 1. Mean (SD) age was 71.7 (13.2) years, with 49.7% females. The majority were White (75.6%) and in the manual social class (59.0%). 62.5% of patients had pre-existing hypertension and 59.9% had had a transient ischemic attack previously. In addition, almost a fifth of patients had preexisting coronary heart disease, atrial fibrillation or diabetes.

**Table 1:** Characteristics of patients with ischemic stroke, South London Stroke Register 1995–2006.

Characteristics	N*	Percentage (unless stated otherwise)
***Socio-demographic factors***		
Age (y), mean (SD)	2590	71.7 (13.2)
Female	1288/2590	49.7
Social class		
*Non-manual*	685/2533	27.0
*Manual*	1495/2533	59.0
*Economically inactive*	353/2533	13.9
Ethnicity		
*White*	1915/2532	75.6
*Black*	485/2532	19.2
*Other*	132/2532	5.2
***Pre-stroke medical conditions***		
Hypertension	1582/2532	62.5
Coronary heart disease	466/2532	18.4
Atrial fibrillation	440/2577	17.1
Diabetes	503/2579	19.5
Transient ischemic attack	1417/2367	59.9
***Pre-stroke lifestyle factors***		
Current smoker	847/2453	34.5
High alcohol consumption	306/1963	15.6
***Classification of ischemic stroke***		
Stroke severity		
*Mild*	1344/2590	51.9
*Severe*	1246/2590	48.1
Oxford classification		
*TACI*	486/2590	18.8
*PACI*	812/2590	31.4
*POCI*	376/2590	14.5
*LACI*	883/2590	34.1
*Unspecified infarct*	33/2590	1.3
TOAST classification		
*Large artery atherosclerosis*	154	-
*Cardioembolism*	420	-
*Small vessel occlusion*	382	-
*Other determined etiology*	53	-
*Undetermined etiology*	504	-
*Missing information*	1077	-

* Denominators vary because of missing information. Denominators are not appropriate for the TOAST classification as this classification was implemented part way through the study period.

With regard to clinical subtypes, the largest categories were LACI (34.1%) and PACI (31.4%). A small proportion (1.3%) had unspecified infarct. As the TOAST etiological classification was introduced part-way through the study period, a substantial number had missing information (1077/2590, 42%). Of those classified, there were 154 with large artery atherosclerosis, 420 with cardioembolism and 382 with small vessel occlusion (Table 1).

The mean daily concentrations of the five outdoor air pollutants are shown in Table 2. Mean (SD) distance from the nearest monitoring station from which the pollution concentration was obtained was in the region of 2.2 (1.2) to 2.6 (1.5) km for the gaseous pollutants and 4.2 (1.8) km for PM_10_.

**Table 2:** Daily concentrations of air pollutants, daily air temperature, national weekly influenza rate and distance from the nearest air pollution monitoring station; South London Stroke Register 1995–2006.

Variable	Mean (SD)	Inter-quartile range	Distance from nearest air pollution monitoring station (km), Mean (SD)
NO_2_ (ppb)	26.4 (10.2)	12.61	2.2 (1.2)
PM_10_ (ug/m^3^)	22.8 (10.5)	10.46	4.2 (1.8)
CO (ppm)	0.6 (0.4)	0.34	2.6 (1.5)
O_3_ (ppb)	15.3 (9.1)	12.75	2.4 (1.3)
SO_2_ (ppb)	3.5 (4.0)	2.89	2.3 (1.4)
Daily temperature (°C)	11.7 (5.6)	8.62	-
Weekly influenza rate (per 100,000 population)	25.2 (34.5)	20.34	-

Table 3 presents adjusted odds ratios associated with an inter-quartile range increase in pollutant concentrations in relation to all ischemic stroke and clinical subtypes. There were a few positive associations. These odds ratios were: 1.07 (1.01–1.13) for NO_2_ at Lag 0 and 1.08 (1.00–1.16) at Lag 5 for all ischemic stroke; 1.11 (1.03–1.20) for CO at Lag 3 for TACI; 1.12 (1.01–1.26) for PM_10_ at Lag 0, 1.23 (1.02–1.50) for O_3_ at Lag 5 and 1.09 (1.00–1.19) for SO_2_ at Lag 0 for POCI; and 1.11 (1.03–1.20) for PM_10_ at Lag 6 for LACI. However, the general pattern overall did not show any consistent positive association with all ischemic stroke or with any clinical subtype. It should also be noted that there were a number of negative associations, especially for CO with POCI.

**Table 3:** Adjusted odds ratios (95% CI) for all ischemic stroke and ischemic stroke classified by clinical (Oxford) subtype associated with an inter-quartile range increase in outdoor air pollutants by lag interval, South London Stroke Register 1995–2006.

Pollutant and Lag	All ischemic stroke	TACI	PACI	POCI	LACI
***PM***_***10***_					
0	1.01 (0.97–1.06)	1.05 (0.95–1.16)	0.93 (0.86–1.02)	1.12 (1.01–1.26) *	1.01 (0.93–1.09)
1	0.98 (0.94–1.03)	0.97 (0.87–1.08)	0.95 (0.87–1.03)	1.01 (0.90–1.14)	1.00 (0.92–1.09)
2	1.00 (0.95–1.04)	0.98 (0.88–1.09)	0.99 (0.91–1.07)	1.02 (0.91–1.15)	0.99 (0.91–1.08)
3	1.01 (0.96–1.05)	1.05 (0.95–1.16)	0.92 (0.84–1.00)	1.09 (0.98–1.22)	1.01 (0.93–1.10)
4	1.01 (0.96–1.05)	1.04 (0.94–1.16)	0.93 (0.85–1.01)	1.04 (0.94–1.17)	1.04 (0.96–1.12)
5	1.00 (0.96–1.05)	0.93 (0.84–1.04)	0.96 (0.88–1.04)	1.07 (0.95–1.19)	1.06 (0.98–1.15)
6	1.01 (0.97–1.06)	0.92 (0.83–1.03)	0.97 (0.89–1.05)	1.02 (0.91–1.15)	1.11 (1.03–1.20) **
0–6	1.02 (0.95–1.10)	1.01 (0.86–1.18)	0.90 (0.79–1.03)	1.12 (0.94–1.32)	1.10 (0.97–1.24)
***NO***_***2***_					
0	1.07 (1.01–1.13) *	1.10 (0.96–1.26)	0.98 (0.88–1.09)	1.16 (0.99–1.36)	1.11 (1.00–1.23)
1	1.02 (0.96–1.08)	1.01 (0.88–1.15)	1.03 (0.92–1.15)	0.93 (0.79–1.10)	1.06 (0.95–1.18)
2	0.98 (0.92–1.05)	1.05 (0.91–1.20)	0.96 (0.85–1.07)	0.89 (0.75–1.05)	1.01 (0.91–1.12)
3	0.99 (0.93–1.05)	1.08 (0.94–1.23)	0.94 (0.84–1.05)	0.96 (0.82–1.13)	1.01 (0.91–1.12)
4	0.97 (0.92–1.04)	1.01 (0.87–1.16)	0.90 (0.80–1.01)	0.93 (0.79–1.09)	1.06 (0.95–1.18)
5	0.97 (0.91–1.03)	0.96 (0.84–1.10)	0.92 (0.82–1.03)	0.95 (0.81–1.11)	1.03 (0.92–1.14)
6	1.00 (0.94–1.07)	0.97 (0.84–1.11)	0.92 (0.82–1.03)	1.05 (0.89–1.23)	1.09 (0.98–1.20)
0–6	1.02 (0.92–1.12)	1.07 (0.87–1.33)	0.89 (0.74–1.06)	0.93 (0.72–1.20)	1.16 (0.99–1.37)
***O***_***3***_					
0	0.99 (0.91–1.06)	0.95 (0.80–1.13)	1.06 (0.93–1.22)	0.98 (0.80–1.20)	0.94 (0.83–1.08)
1	1.00 (0.93–1.08)	0.94 (0.79–1.12)	0.99 (0.86–1.14)	1.03 (0.84–1.26)	1.05 (0.92–1.20)
2	1.03 (0.95–1.11)	0.83 (0.69–0.99) *	1.10 (0.96–1.27)	1.18 (0.96–1.44)	1.03 (0.90–1.17)
3	1.04 (0.97–1.12)	0.94 (0.79–1.12)	1.09 (0.95–1.25)	1.09 (0.89–1.33)	1.03 (0.90–1.17)
4	1.04 (0.97–1.13)	1.02 (0.85–1.21)	1.06 (0.93–1.22)	1.11 (0.90–1.35)	0.98 (0.86–1.12)
5	1.08 (1.00–1.16) *	1.03 (0.87–1.23)	1.13 (0.99–1.30)	1.23 (1.02–1.50) *	0.99 (0.87–1.12)
6	1.00 (0.93–1.08)	0.96 (0.80–1.14)	1.12 (0.98–1.28)	1.07 (0.88–1.31)	0.88 (0.77–1.01)
0–6	1.08 (0.96–1.21)	0.92 (0.70–1.20)	1.18 (0.96–1.45)	1.21 (0.90–1.65)	1.01 (0.83–1.23)
***CO***					
0	0.99 (0.95–1.03)	1.07 (0.99–1.16)	0.92 (0.84–1.00) *	0.99 (0.90–1.10)	1.00 (0.93–1.07)
1	0.96 (0.92–1.00)	1.02 (0.94–1.11)	0.91 (0.84–0.99) *	0.91 (0.81–1.01)	0.99 (0.92–1.06)
2	0.95 (0.91–0.99) *	1.04 (0.96–1.14)	0.90 (0.83–0.98) *	0.90 (0.80–1.01)	0.97 (0.90–1.04)
3	0.99 (0.95–1.03)	1.11 (1.03–1.20) **	0.93 (0.86–1.01)	0.90 (0.80–1.02)	0.99 (0.92–1.06)
4	0.98 (0.94–1.02)	1.05 (0.97–1.15)	0.91 (0.84–1.00) *	0.92 (0.83–1.03)	1.02 (0.95–1.09)
5	0.98 (0.94–1.02)	1.03 (0.95–1.11)	0.90 (0.82–0.98) *	0.94 (0.85–1.04)	1.03 (0.96–1.10)
6	0.98 (0.94–1.02)	1.01 (0.93–1.10)	0.94 (0.87–1.02)	0.91 (0.81–1.02)	1.04 (0.97–1.11)
0–6	0.94 (0.88–1.01)	1.13 (0.99–1.29)	0.81 (0.71–0.93) **	0.83 (0.70–0.98) *	1.01 (0.90–1.13)
***SO***_***2***_					
0	1.01 (0.98–1.05)	1.03 (0.97–1.10)	0.95 (0.89–1.02)	1.09 (1.00–1.19) *	1.01 (0.95–1.08)
1	1.00 (0.97–1.04)	1.01 (0.94–1.09)	0.98 (0.92–1.05)	0.99 (0.90–1.09)	1.01 (0.95–1.08)
2	1.00 (0.96–1.03)	1.01 (0.93–1.09)	0.96 (0.90–1.02)	1.00 (0.92–1.09)	1.02 (0.96–1.08)
3	0.99 (0.95–1.03)	1.01 (0.94–1.09)	0.97 (0.91–1.04)	1.05 (0.97–1.13)	0.95 (0.89–1.02)
4	0.98 (0.94–1.01)	1.01 (0.93–1.09)	0.93 (0.87–1.00)	0.97 (0.89–1.06)	0.99 (0.93–1.06)
5	1.01 (0.98–1.04)	0.98 (0.90–1.06)	0.99 (0.93–1.05)	1.04 (0.97–1.13)	1.03 (0.97–1.09)
6	1.02 (0.99–1.05)	1.02 (0.95–1.10)	1.01 (0.95–1.07)	1.02 (0.93–1.12)	1.03 (0.97–1.09)
0–6	1.01 (0.95–1.07)	1.04 (0.91–1.17)	0.94 (0.85–1.05)	1.05 (0.92–1.21)	1.02 (0.93–1.13)

* p<0.05

** p<0.01

Table 4 presents adjusted odds ratios associated with an inter-quartile range increase in pollutant concentrations in relation to severity of ischemic stroke and etiological subtype. There were a number of positive associations. These odds ratios were: 1.14 (1.03–1.26) for O_3_ at Lag 5 for mild ischemic stroke; 1.10 (1.01–1.19) for NO_2_ at Lag 0 for severe ischemic stroke; 1.28 (1.07–1.53) at Lag 0 and 1.24 (1.01–1.52) at Lag 3 for PM_10_ and 1.35 (1.02–1.80) at Lag 1 for NO_2_ in relation to large artery atherosclerosis; and 1.25 (1.04–1.50) at Lag 4 and 1.36 (1.13–1.63) at Lag 5 for O_3_ and 1.16 (1.01–1.35) at Lag 1 for CO in relation to cardioembolism. With regard to small vessel occlusion, the adjusted odds ratios were: 1.14 (1.00–1.29) at Lag 3 and 1.21 (1.07–1.36) at Lag 6 for PM_10_; 1.21 (1.00–1.46) at Lag 3, 1.21 (1.01–1.45) at Lag 5, 1.26 (1.05–1.52) at Lag 6 and 1.51 (1.12–2.02) at Lag 0–6 for NO_2_; and 1.30 (1.12–1.51) at Lag 5 for SO_2_. Overall, there were no consistent associations between pollutants and severity or etiological subtype, possibly with the exception of NO_2_ and small vessel occlusion where there was a suggestion of an association.

**Table 4:** Adjusted odds ratios (95% CI) for mild and severe ischemic stroke and ischemic stroke classified by etiological (TOAST) subtype associated with an inter-quartile range increase in outdoor air pollutants by lag interval, South London Stroke Register 1995–2006.

Pollutant and Lag	Mild ischemic stroke	Severe ischemic stroke	Large artery atherosclerosis	Cardioembolism	Small vessel occlusion
***PM***_***10***_					
0	0.96 (0.90–1.03)	1.06 (1.00–1.13)	1.28 (1.07–1.53) **	1.09 (0.96–1.23)	0.99 (0.86–1.14)
1	0.97 (0.91–1.04)	0.99 (0.93–1.06)	1.20 (0.97–1.48)	1.08 (0.95–1.23)	0.94 (0.81–1.08)
2	0.98 (0.92–1.05)	1.01 (0.95–1.08)	1.14 (0.92–1.41)	1.02 (0.89–1.17)	1.03 (0.90–1.18)
3	1.01 (0.95–1.08)	1.00 (0.94–1.07)	1.24 (1.01–1.52) *	0.89 (0.77–1.03)	1.14 (1.00–1.29) *
4	0.97 (0.90–1.03)	1.05 (0.98–1.11)	1.03 (0.82–1.30)	1.02 (0.89–1.16)	1.09 (0.96–1.23)
5	1.01 (0.95–1.08)	1.00 (0.94–1.07)	0.80 (0.62–1.05)	0.99 (0.87–1.13)	1.10 (0.97–1.25)
6	1.04 (0.98–1.11)	0.99 (0.92–1.05)	0.92 (0.72–1.17)	0.96 (0.84–1.10)	1.21 (1.07–1.36) **
0–6	0.99 (0.90–1.10)	1.05 (0.95–1.16)	1.28 (0.91–1.79)	1.03 (0.84–1.26)	1.19 (0.98–1.45)
***NO***_***2***_					
0	1.04 (0.96–1.13)	1.10 (1.01–1.19) *	1.22 (0.92–1.62)	1.07 (0.89–1.28)	1.09 (0.90–1.31)
1	1.01 (0.92–1.10)	1.03 (0.95–1.13)	1.35 (1.02–1.80) *	1.07 (0.89–1.27)	1.04 (0.86–1.26)
2	0.96 (0.88–1.04)	1.01 (0.93–1.10)	1.15 (0.88–1.51)	1.01 (0.85–1.20)	1.12 (0.93–1.35)
3	0.98 (0.90–1.07)	1.00 (0.91–1.09)	1.09 (0.82–1.45)	0.92 (0.77–1.10)	1.21 (1.00–1.46) *
4	0.93 (0.85–1.02)	1.02 (0.93–1.11)	1.11 (0.83–1.49)	0.88 (0.74–1.06)	1.14 (0.95–1.37)
5	0.96 (0.88–1.04)	0.98 (0.90–1.07)	1.09 (0.81–1.46)	0.84 (0.70–1.01)	1.21 (1.01–1.45) *
6	1.00 (0.92–1.09)	1.01 (0.92–1.10)	1.07 (0.79–1.45)	1.01 (0.85–1.21)	1.26 (1.05–1.52) *
0–6	0.97 (0.84–1.11)	1.07 (0.93–1.23)	1.44 (0.92–2.24)	0.96 (0.72–1.27)	1.51 (1.12–2.02) **
***O***_***3***_					
0	0.99 (0.89–1.10)	0.98 (0.88–1.10)	1.01 (0.75–1.36)	0.91 (0.76–1.10)	0.93 (0.77–1.14)
1	1.04 (0.94–1.16)	0.96 (0.86–1.08)	0.96 (0.71–1.31)	1.03 (0.86–1.24)	1.06 (0.87–1.29)
2	1.07 (0.96–1.19)	0.98 (0.88–1.10)	1.03 (0.76–1.41)	1.15 (0.96–1.38)	0.96 (0.80–1.17)
3	1.03 (0.93–1.15)	1.05 (0.94–1.17)	1.07 (0.79–1.46)	1.17 (0.98–1.40)	0.94 (0.77–1.14)
4	1.08 (0.97–1.20)	1.00 (0.90–1.12)	1.08 (0.80–1.46)	1.25 (1.04–1.50) *	1.08 (0.89–1.31)
5	1.14 (1.03–1.26) *	1.02 (0.91–1.13)	1.23 (0.91–1.66)	1.36 (1.13–1.63) **	0.88 (0.73–1.07)
6	1.02 (0.92–1.13)	0.97 (0.87–1.09)	0.90 (0.66–1.21)	1.09 (0.91–1.31)	0.86 (0.71–1.04)
0–6	1.14 (0.97–1.33)	1.01 (0.86–1.20)	1.09 (0.69–1.74)	1.25 (0.95–1.66)	0.96 (0.72–1.29)
***CO***					
0	0.95 (0.89–1.01)	1.02 (0.97–1.08)	1.00 (0.79–1.27)	1.04 (0.89–1.21)	0.94 (0.78–1.13)
1	0.92 (0.86–0.98) *	0.99 (0.94–1.05)	1.01 (0.79–1.29)	1.16 (1.01–1.35) *	1.00 (0.84–1.19)
2	0.94 (0.88–1.00) *	0.97 (0.91–1.03)	0.95 (0.75–1.22)	1.07 (0.92–1.23)	1.03 (0.88–1.22)
3	0.96 (0.90–1.02)	1.01 (0.95–1.06)	1.06 (0.84–1.35)	0.93 (0.79–1.09)	1.12 (0.95–1.31)
4	0.96 (0.90–1.02)	1.00 (0.94–1.05)	1.13 (0.89–1.42)	0.87 (0.74–1.02)	0.99 (0.82–1.18)
5	0.96 (0.91–1.03)	0.99 (0.94–1.05)	1.15 (0.91–1.45)	0.91 (0.78–1.07)	1.08 (0.91–1.28)
6	0.97 (0.92–1.04)	0.99 (0.93–1.05)	1.12 (0.88–1.44)	0.85 (0.72–1.01)	1.11 (0.94–1.32)
0–6	0.88 (0.80–0.97) *	1.00 (0.91–1.09)	1.14 (0.80–1.65)	0.98 (0.77–1.26)	1.06 (0.80–1.41)
***SO***_***2***_					
0	0.99 (0.94–1.05)	1.03 (0.98–1.08)	0.93 (0.69–1.24)	0.99 (0.83–1.17)	1.11 (0.95–1.30)
1	0.99 (0.94–1.04)	1.01 (0.97–1.06)	1.13 (0.90–1.42)	1.12 (0.96–1.30)	1.03 (0.87–1.22)
2	0.98 (0.93–1.03)	1.01 (0.96–1.06)	1.10 (0.85–1.41)	0.98 (0.83–1.15)	1.04 (0.88–1.23)
3	0.99 (0.94–1.05)	0.99 (0.94–1.04)	1.11 (0.86–1.43)	0.87 (0.73–1.03)	0.93 (0.78–1.12)
4	0.95 (0.90–1.00)	1.00 (0.95–1.05)	1.07 (0.83–1.37)	0.92 (0.78–1.10)	0.99 (0.83–1.17)
5	1.03 (0.98–1.08)	0.99 (0.95–1.04)	1.01 (0.75–1.35)	0.90 (0.76–1.07)	1.30 (1.12–1.51) ***
6	1.02 (0.97–1.07)	1.02 (0.98–1.07)	1.00 (0.76–1.31)	0.85 (0.71–1.01)	1.14 (0.96–1.34)
0–6	0.99 (0.91–1.07)	1.02 (0.95–1.11)	1.13 (0.75–1.72)	0.89 (0.67–1.17)	1.18 (0.90–1.55)

* p<0.05

** p<0.01

*** p<0.001

Table 5 shows the adjusted odds ratios for all ischemic stroke associated with an inter-quartile range increase in air pollutants in relation to sociodemographic factors, preexisting medical conditions and pre-stroke lifestyle factors. There were three positive associations but also four negative associations. Overall, there was little to suggest that preexisting risk factors increased susceptibility to the adverse effects of air pollutants in terms of ischemic stroke risk.

**Table 5:** Adjusted odds ratios (95% CI) for all ischemic stroke associated with an inter-quartile range increase in outdoor air pollutants (0–6 day average) by sociodemographic factors, lifestyle-related risk factors and pre-existing medical conditions, South London Stroke Register 1995–2006.

Characteristics	PM_10_	NO_2_	O_3_	CO	SO_2_
***Socio-demographic factors***					
Age					
*<70 years*	0.95 (0.84–1.06)	0.95 (0.81–1.11)	1.11 (0.92–1.33)	0.89 (0.79–1.00) *	1.01 (0.92–1.11)
*> = 70years*	1.07 (0.98–1.16)	1.06 (0.93–1.20)	1.06 (0.91–1.23)	0.97 (0.89–1.05)	1.01 (0.93–1.08)
Gender					
*Male*	1.03 (0.93–1.13)	0.98 (0.86–1.13)	1.07 (0.91–1.26)	0.91 (0.83–1.00)	1.01 (0.93–1.09)
*Female*	1.02 (0.92–1.12)	1.05 (0.92–1.20)	1.09 (0.92–1.28)	0.97 (0.89–1.06)	1.01 (0.93–1.09)
Social Class					
*Non-manual*	1.01 (0.89–1.16)	0.94 (0.78–1.15)	1.16 (0.93–1.45)	0.91 (0.80–1.03)	1.01 (0.90–1.13)
*Manual*	1.00 (0.91–1.10)	1.02 (0.90–1.16)	1.08 (0.92–1.25)	0.92 (0.84–1.00)	0.98 (0.91–1.05)
*Economically inactive*	1.13 (0.93–1.37)	1.05 (0.81–1.37)	1.00 (0.73–1.37)	1.11 (0.94–1.31)	1.15 (0.99–1.32)
Ethnicity					
*White*	1.01 (0.93–1.10)	0.97 (0.87–1.08)	1.10 (0.96–1.26)	0.93 (0.86–1.00)	1.00 (0.94–1.07)
*Black*	0.98 (0.83–1.16)	1.11 (0.89–1.40)	1.06 (0.81–1.38)	0.96 (0.81–1.14)	1.01 (0.88–1.16)
*Other*	1.27 (0.94–1.73)	1.47 (0.93–2.31)	0.91 (0.55–1.50)	1.07 (0.79–1.45)	1.17 (0.90–1.53)
***Pre-stroke medical conditions***					
Hypertension					
*No*	1.01 (0.89–1.14)	0.97 (0.82–1.14)	0.95 (0.79–1.16)	0.96 (0.86–1.07)	1.05 (0.95–1.16)
*Yes*	1.02 (0.94–1.12)	1.03 (0.91–1.16)	1.16 (1.00–1.34) *	0.93 (0.85–1.01)	0.99 (0.92–1.06)
Coronary heart disease					
*No*	1.00 (0.93–1.09)	0.97 (0.87–1.09)	1.07 (0.94–1.22)	0.92 (0.85–0.99) *	1.00 (0.94–1.07)
*Yes*	1.06 (0.92–1.23)	1.20 (0.97–1.48)	1.03 (0.79–1.35)	1.01 (0.88–1.17)	1.04 (0.92–1.18)
Atrial fibrillation					
*No*	1.06 (0.98–1.14)	1.09 (0.98–1.21)	1.03 (0.91–1.17)	0.94 (0.87–1.01)	1.04 (0.98–1.11)
*Yes*	0.87 (0.73–1.03)	0.74 (0.58–0.93) *	1.36 (1.02–1.79) *	0.96 (0.84–1.10)	0.86 (0.74–0.99) *
Diabetes					
*No*	1.02 (0.95–1.10)	1.04 (0.94–1.16)	1.07 (0.94–1.22)	0.96 (0.89–1.03)	1.01 (0.95–1.08)
*Yes*	1.01 (0.86–1.19)	0.90 (0.71–1.13)	1.10 (0.85–1.42)	0.87 (0.73–1.03)	0.98 (0.86–1.12)
Transient ischemic attack					
*No*	1.01 (0.89–1.14)	0.97 (0.82–1.14)	0.95 (0.79–1.16)	0.96 (0.86–1.07)	1.05 (0.95–1.16)
*Yes*	1.04 (0.95–1.14)	1.06 (0.94–1.20)	1.11 (0.96–1.30)	0.93 (0.85–1.01)	1.00 (0.93–1.08)
***Pre-stroke lifestyle factors***					
Current smoker					
*No*	1.08 (0.99–1.18)	1.05 (0.93–1.18)	1.18 (1.02–1.36) *	0.94 (0.87–1.02)	1.03 (0.96–1.11)
*Yes*	0.91 (0.80–1.03)	0.89 (0.75–1.07)	0.94 (0.76–1.15)	0.94 (0.84–1.06)	0.96 (0.87–1.06)
High alcohol consumption					
*No*	1.06 (0.98–1.15)	1.04 (0.93–1.17)	1.07 (0.93–1.24)	0.95 (0.88–1.03)	1.03 (0.97–1.10)
*Yes*	0.89 (0.72–1.10)	0.87 (0.65–1.15)	1.06 (0.76–1.49)	0.92 (0.76–1.10)	0.99 (0.84–1.16)

* p<0.05

Sensitivity analyses using control days sampled from the same calendar month instead of the same season and using all pollution concentrations for a pollutant obtained from the same monitoring station for an individual patient gave similar overall results. (Comparison of odds ratios calculated using control days selected from the same month with odds ratios calculated using control days selected from the same season is shown in S1 Table.)

## Discussion

We investigated whether there was any association between short term variation in outdoor air pollutants and acute ischemic stroke, particularly in relation to clinical and etiological subtypes and severity. We found associations at various lag times with several of the pollutants examined. Overall, however, there was no consistent pattern between short term exposure and increased risk of ischemic stroke subtypes or severity. The possible exception was the suggestion that NO_2_ exposure might be associated with stroke caused by cerebrovascular small vessel disease, which included an adjusted odds ratio of 1.51 (1.12–2.02) associated with an inter-quartile range increase in the lag 0–6 day average for NO_2_.

We also examined if pre-existing risk factors for stroke, which included sociodemographic factors, lifestyle-related risk factors and pre-existing medical conditions, increased susceptibility to air pollution related ischemic stroke. There was little to suggest that these pre-existing risk factors increased susceptibility to the adverse effects of air pollution on ischemic stroke risk.

Few previous studies have investigated the association between air pollution and ischemic stroke subtypes. Henrotin et al examined the effects of PM_10_, NO_2_, CO, SO_2_ and O_3_ at lags of 0 to 3 days in 1487 patients with ischemic stroke in Dijon, France [9]. Their only significant finding was an association between O_3_ and ischemic stroke at a 1-day lag. Analysis by gender found that this was only significant for men. The authors then examined associations with ischemic stroke etiological subtypes in men using a 1-day lag and found an association between O_3_ and large vessel disease stroke but not lacunar stroke or cardioembolic stroke.

O’Donnell et al investigated the association between PM_2.5_ and acute ischemic stroke in 9202 hospitalized patients using data from the Registry of the Canadian Stroke Network [16]. Their primary analysis used PM_2.5_ values 0–47 hours prior to stroke onset. Sensitivity analyses included lag periods of up to 7 days. They found no overall association between PM_2.5_ and ischemic stroke risk. However, there were non-significant positive associations with large vessel disease stroke and small vessel disease stroke but a significant inverse association with cardioembolic stroke.

Wellenius et al investigated associations between PM_2.5_, black carbon, sulfate particles, NO_2_, CO and O_3_ and short-term risk of ischemic stroke in 1705 patients admitted from the Boston area of USA [5]. They investigated successive 24-hour lag periods from <24 hours to 72 to <96 hours. They found significant positive associations between PM_2.5_, black carbon and NO_2_ and ischemic stroke for the <24 hour lag period but not for longer lag periods. There were no significant associations with the other three pollutants examined. PM_2.5_ using the <24 hour lag was examined with respect to etiological subtypes and positive associations were found with large artery stroke and small vessel stroke but not with cardioembolic stroke.

Corea et al investigated associations between PM_10_, CO, NO, NO_2_, SO_2_, benzene and O_3_ and stroke subtypes in patients admitted acutely to a stroke unit in Mantua, Italy [17]. The analysis was confined to 781 patients (567 with ischemic stroke) living within 10 km of one of the urban air pollution monitoring stations included in the study. They used the Oxford clinical classification system in addition to the etiological classification. No significant associations were observed in analyses using all ischemic stroke cases combined. In the analysis of subtypes, there was a significant association between PM_10_ and LACI. In addition, the authors reported an association between PM_10_ and TACI which was significant in men only. There were no significant associations with any of the other pollutants examined.

One previous study has investigated if the association between short-term exposure to air pollution and ischemic stroke varies by severity of the resulting stroke. Andersen et al examined the effects of short-term exposure to ultrafine particles (particles <0.1um in diameter), PM_10_, NO_x_ and CO on hospital admissions for stroke [10]. Data were analyzed on 6798 ischemic and 687 hemorrhagic strokes. 61% of strokes were classified as mild and 39% as severe. The authors found significant positive associations between ultrafine particles and NO_x_ and mild, but not severe, ischemic stroke with strongest associations observed at a 4-day lag.

With regard to susceptibility, Wellenius et al found no evidence to suggest that the association between PM_2.5_ and ischemic stroke was modified by preexisting diabetes, atrial fibrillation, hypertension, a history of stroke or TIA or older age [5]. Henrotin et al observed an association between 0_3_ and ischemic stroke only in men and in addition found that the association was stronger in men with preexisting cardiovascular risk factors including smoking, dyslipidemia and hypertension [9]. O’Donnell et al found that whilst there was no significant association between PM_2.5_ and ischemic stroke overall, there was a significant association in patients with pre-existing diabetes [16]. They found no evidence to indicate that other factors, including atrial fibrillation, hypertension, prior stroke or TIA, smoking and gender, modified the association. Villeneuve et al found that the association between NO_2_ and ischemic stroke was stronger in patients with a history of stroke or heart disease or on medication for diabetes [11].

Comparison of the concentrations of pollutants examined in our study with concentrations observed in the studies discussed above was restricted by the information provided on those studies and the units of measurement used. The mean PM_10_ concentration of 22.8ug/m^3^ in our study was similar to the mean of 21.1ug/m^3^ reported by Henrotin et al for Dijon but lower than the mean of 27.1ug/m^3^ reported by Andersen et al for Copenhagen [9,10]. However, the mean CO concentration in our study of 0.6ppm was higher than the mean of 0.27ppm for Copenhagen [10]. Our observed means of 3.5ppb for SO_2_, 26.4ppb for NO_2_ and 0.6ppm for CO were approximately double the mean concentrations of SO_2_ (1.5ppb), NO_2_ (12.4ppb) and CO (0.3ppm) reported by Villeneuve et al for Edmonton, Canada [11].

Some of the previous studies suggest that we ought to have observed clearer patterns in the associations between air pollutants and ischemic stroke subtypes. Potential limitations of our study might explain why we found little consistent evidence, possibly with the exception of NO_2_ and cerebrovascular small vessel disease. Our study, which was based on 2590 incident stroke cases, may have lacked power to detect associations. However, whilst the study by O’Donnell et al was based on a substantially larger number of ischemic strokes (9209 cases), the studies by Henrotin et al, Wellenius et al and Corea et al were based on comparable numbers of ischemic stroke cases (1487, 1705 and 567 cases respectively) [5,9,16,17].

A second possibility is exposure misclassification. Mean (SD) distances from residential postcode centroids of stroke patients in our study to the nearest monitoring stations from which the measurements were obtained were in the region of 2.2 (1.2) to 2.6 (1.5) km for the gaseous pollutants and 4.2 (1.8) km for PM_10_. Despite limitations regarding comparability of our distance data with studies reviewed above, it is unlikely that exposure misclassification in our study due to distance from monitoring stations was substantially greater than in the studies reviewed which stipulated maximum distances ranging from 10 to 50 km [5,16,17].

There may have been error in classification of clinical and etiological subtypes in our study. However, the South London Stroke Register used standardized methods similar to those used in the other studies and misclassification of stroke is unlikely to have been substantially different from that in the other studies. Because of the relatively small number of strokes we examined, we increased power by using control days sampled from within the same season rather than the same month. Although not frequently used, sampling from the same season is a recognized approach [21,22]. In our sensitivity analysis, we used controls in the same month and this did not change the overall conclusions.

A further issue that needs to be considered is publication bias. It is possible that negative studies are less likely to be published and analyses showing null associations are less likely to be reported in published studies. Evidence of publication bias was observed in a meta-analysis examining short-term effects of particulate matter on risk of stroke [3].

In summary, we found little consistent evidence of association between a range of outdoor air pollutants and ischemic stroke subtypes and severity, possibly with the exception of an association between NO_2_ and stroke caused by cerebrovascular small vessel disease. In addition, we found no evidence to suggest that pre-existing risk factors increased susceptibility to the adverse effects of air pollution on ischemic stroke risk. Further studies are needed to examine these associations. Such studies will enhance pathophysiological understanding of the mechanisms by which air pollution could cause ischemic stroke.

## Supporting Information

S1 Table Comparison of same month and same season controls. (DOC)
