# Supplementary material for: Air Pollution and Subtypes, Severity and Vulnerability to Ischemic Stroke—A Population Based Case-Crossover Study
Source: PLoS One. 2016 Jun 30;11(6):e0158556. doi: 10.1371/journal.pone.0158556 (PMC4928841; doi:10.1371/journal.pone.0158556)
Supplement: S1 Table — (DOC) [file pone.0158556.s001.doc]

**S1 Table. Comparison of same month and same season controls.**

| **Ischemic stroke category** | **Pollutant** | **Control days from same month** | **Control days from same season** |
| --- | --- | --- | --- |
| ***All ischemic stroke*** |  |  |  |
|  | PM_10_ | 1.02 (0.94 - 1.11) | 1.02 (0.95 - 1.10) |
|  | NO_2_ | 0.98 (0.88 - 1.10) | 1.02 (0.92 - 1.12) |
|  | O_3_ | 1.09 (0.94 - 1.26) | 1.08 (0.96 - 1.21) |
|  | CO | 0.97 (0.90 - 1.05) | 0.94 (0.88 - 1.01) |
|  | SO_2_ | 1.01 (0.95 - 1.08) | 1.01 (0.95 - 1.07) |
| ***TACI*** |  |  |  |
|  | PM_10_ | 1.03 (0.85 - 1.25) | 1.01 (0.86 - 1.18) |
|  | NO_2_ | 1.08 (0.84 - 1.38) | 1.07 (0.87 - 1.33) |
|  | O_3_ | 0.95 (0.67 - 1.33) | 0.92 (0.70 - 1.20) |
|  | CO | 1.17 (0.99 - 1.38) | 1.13 (0.99 - 1.29) |
|  | SO_2_ | 1.10 (0.94 - 1.28) | 1.04 (0.91 - 1.17) |
| ***PACI*** |  |  |  |
|  | PM_10_ | 0.91 (0.78 - 1.06) | 0.90 (0.79 - 1.03) |
|  | NO_2_ | 0.89 (0.72 - 1.09) | 0.89 (0.74 - 1.06) |
|  | O_3_ | 1.14 (0.87 - 1.48) | 1.18 (0.96 - 1.45) |
|  | CO | 0.83 (0.71 - 0.96)* | 0.81 (0.71 - 0.93)** |
|  | SO_2_ | 0.96 (0.85 - 1.08) | 0.94 (0.85 - 1.05) |
| ***POCI*** |  |  |  |
|  | PM_10_ | 1.01 (0.83 - 1.22) | 1.12 (0.94 - 1.32) |
|  | NO_2_ | 0.73 (0.54 - 0.98)* | 0.93 (0.72 - 1.20) |
|  | O_3_ | 1.32 (0.91 - 1.93) | 1.21 (0.90 - 1.65) |
|  | CO | 0.79 (0.65 - 0.97)* | 0.83 (0.70 - 0.98)* |
|  | SO_2_ | 0.97 (0.83 - 1.13) | 1.05 (0.92 - 1.21) |
| ***LACI*** |  |  |  |
|  | PM_10_ | 1.11 (0.96 - 1.28) | 1.10 (0.97 - 1.24) |
|  | NO_2_ | 1.16 (0.96 - 1.41) | 1.16 (0.99 - 1.37) |
|  | O_3_ | 1.01 (0.78 - 1.29) | 1.01 (0.83 - 1.23) |
|  | CO | 1.11 (0.97 - 1.27) | 1.01 (0.90 - 1.13) |
|  | SO_2_ | 1.04 (0.93 - 1.17) | 1.02 (0.93 - 1.13) |
| ***Mild ischemic stroke*** |  |  |  |
|  | PM_10_ | 0.99 (0.88 - 1.11) | 0.99 (0.90 - 1.10) |
|  | NO_2_ | 0.90 (0.77 - 1.06) | 0.97 (0.84 - 1.11) |
|  | O_3_ | 1.11 (0.91 - 1.37) | 1.14 (0.97 - 1.33) |
|  | CO | 0.91 (0.81 - 1.02) | 0.88 (0.80 - 0.97)* |
|  | SO_2_ | 1.01 (0.91 - 1.11) | 0.99 (0.91 - 1.07) |
| ***Severe ischemic stroke*** |  |  |  |
|  | PM_10_ | 1.05 (0.94 - 1.18) | 1.05 (0.95 - 1.16) |
|  | NO_2_ | 1.07 (0.91 - 1.25) | 1.07 (0.93 - 1.23) |
|  | O_3_ | 1.06 (0.86 - 1.30) | 1.01 (0.86 - 1.20) |
|  | CO | 1.03 (0.93 - 1.15) | 1.00 (0.91 - 1.09) |
|  | SO_2_ | 1.02 (0.93 - 1.11) | 1.02 (0.95 - 1.11) |
| ***Large artery atherosclerosis*** |  |  |  |
|  | PM_10_ | 1.33 (0.89 - 1.99) | 1.28 (0.91 - 1.79) |
|  | NO_2_ | 1.22 (0.73 - 2.06) | 1.44 (0.92 - 2.24) |
|  | O_3_ | 1.34 (0.74 - 2.42) | 1.09 (0.69 - 1.74) |
|  | CO | 1.11 (0.75 - 1.63) | 1.14 (0.80 - 1.65) |
|  | SO_2_ | 1.20 (0.71 - 2.01) | 1.13 (0.75 - 1.72) |
| ***Cardioembolism*** |  |  |  |
|  | PM_10_ | 1.02 (0.80 - 1.29) | 1.03 (0.84 - 1.26) |
|  | NO_2_ | 0.91 (0.65 - 1.26) | 0.96 (0.72 - 1.27) |
|  | O_3_ | 1.21 (0.84 - 1.74) | 1.25 (0.95 - 1.66) |
|  | CO | 1.02 (0.77 - 1.35) | 0.98 (0.77 - 1.26) |
|  | SO_2_ | 0.99 (0.70 - 1.38) | 0.89 (0.67 - 1.17) |
| ***Small vessel occlusion*** |  |  |  |
|  | PM_10_ | 1.11 (0.88 - 1.40) | 1.19 (0.98 - 1.45) |
|  | NO_2_ | 1.24 (0.88 - 1.73) | 1.51 (1.12 - 2.02)** |
|  | O_3_ | 1.07 (0.73 - 1.56) | 0.96 (0.72 - 1.29) |
|  | CO | 1.14 (0.83 - 1.56) | 1.06 (0.80 - 1.41) |
|  | SO_2_ | 0.99 (0.71 - 1.38) | 1.18 (0.90 - 1.55) |

Comparison of odds ratios calculated using control days selected from the same month with odds ratios calculated using control days selected from the same season. Adjusted odds ratios (95% CI) for ischemic stroke categories associated with an interquartile range increase in outdoor air pollutants (0-6 day average), South London Stroke Register 1995-2006.

* p<0.05 ** p<0.01
